# Sacred space: a qualitative interpretive meta-synthesis of women’s experiences of supportive birthing environments

**DOI:** 10.1186/s12884-024-06544-6

**Published:** 2024-05-15

**Authors:** December Maxwell, Sarah R. Leat, Toni Gallegos, Regina T. Praetorius

**Affiliations:** 1 The University of Oklahoma, Norman, OK USA; 2 The University of Memphis, Memphis, TN USA; 3 The University of Texas at Arlington, Arlington, TX USA

**Keywords:** Qualitative Interpretive Meta-Synthesis (QIMS), Birthing process, Becoming a mother, Built environment

## Abstract

**Background:**

In the United States there are roughly three million births a year, ranging from cesarean to natural births. A major aspect of the birthing process is related to the healing environment, and how that helps or harms healing for the mother and child. Using the theoretical framework, *Theory of Supportive Care Settings* (TSCS), this study aimed to explore what is necessary to have a safe and sacred healing environment for mothers.

**Method:**

This study utilized an updated Qualitative Interpretive Meta-synthesis (QIMS) design called QIMS-DTT [deductive theory testing] to answer the research question, *What are mother’s experiences of environmental factors contributing to a supportive birthing environment within healthcare settings?*

**Results:**

Key terms were run through multiple databases, which resulted in 5,688 articles. After title and abstract screening, 43 were left for full-text, 12 were excluded, leaving 31 to be included in the final QIMS. Five main themes emerged from analysis: 1) Service in the environment, 2) Recognizing oneself within the birthing space, 3) Creating connections with support systems, 4) Being welcomed into the birthing space, and 5) Feeling safe within the birthing environment.

**Conclusions:**

Providing a warm and welcoming birth space is crucial for people who give birth to have positive experiences. Providing spaces where the person can feel safe and supported allows them to find empowerment in the situation where they have limited control.

## Introduction

In 2021, there were 3,664,292 births in the United States. Of those birth, 98.3% took place in hospitals [1]. In hospital settings, medical interventions such as induction of labor, cesarean sections, and the use of instruments like forceps or vacuum extractors may be more common [2]. These interventions can carry risks such as increased likelihood of complications for both the birthing person and the baby [2, 3]. Some women may feel stressed or anxious in a hospital setting, which could potentially slow down labor or lead to other complications. This stress can be due to various factors such as unfamiliar surroundings, medical procedures, or concerns about interventions [2]. In a hospital setting, decisions about the birth process may be influenced by hospital policies, medical protocols, and the preferences of healthcare providers, potentially leading to a loss of autonomy for the birthing person in decision-making about their own birth experience [4]. The experience of giving birth in a hospital, especially if it involves unexpected interventions or complications, can contribute to postpartum depression or anxiety in some women [5]. Hospital routines and policies may not always be conducive to establishing breastfeeding immediately after birth, which can lead to challenges in breastfeeding initiation and continuation [6].

Birthing requires healing and a supportive environment at every stage of the birthing process, consisting of holistic support and agency [7]. This involves “constant emotional, physical, spiritual, and psychosocial” support [8]. Experiencing birthing trauma has shown to result in postpartum post-traumatic stress disorder (P-PTSD) and postpartum depression (PPD) [9–11]. Likewise, disempowering births can have long term impacts of maternal self-esteem [12, 13]. Maternal mental health issues have resulted in numerous public health concerns, specifically regarding the decreased safety and negative health outcomes that the infant faces [14, 15]. Postpartum mental health disorders can also have lasting impacts on family outcomes [16, 17]. As such, understanding how to improve the birth experience has the potential to reduce postpartum mental health issues, as well as reduce maternal morbidities, which can improve outcomes for both mother and child.

Of note is the influence of the built environment on healing. Given that thoughtfully designed healthcare facilities can influence the amount of privacy and control a patient perceives [18], the built environment plays an integral part in healing. Ample daylight, thermal comfort, color, and noise control all contribute to environmental healing within a hospital [19]. Furthermore, patient health outcomes have been linked to the built environment of hospitals in multiple studies [13, 20, 21]. More specific to birthing, women have indicated that perceived hominess and control in the environment relate to their birthing experience [20, 22, 23].

Control over the birthing environment, including comfort and perceived healing also have mental health impacts for birthing mothers, and the birth environment can have an impact on the mother’s perception of the birth which in turn can influence maternal mental health outcomes [24, 25]. Given that approximately 1 in 7 mothers will experience postpartum depression (PPD) in the United States [26, 27] and 0.05%-60% of mothers will experience PPD globally [28, 29], understanding the impact of birthing environment on maternal morbidities and mental health can create holistic approaches to birthing environment design.

Given the impacts of the birthing environment on maternal mental health, learning what is necessary to have a safe and sacred healing environment for mothers is an important endeavor and the purpose of this qualitative interpretive meta-synthesis (QIMS). A QIMS is a method that is specific to the social work field. It was created to review and analyze qualitative data to identify and synthesize themes surrounding different phenomena found in existing qualitative research [30]. QIMS has previously been used to synthesize existing data regarding social justice concerns around minority police encounters [31] and children’s exposure to intimate partner violence [32]. Concerning the topic of birthing and motherhood, one QIMS explored marginalized women’s experiences of postpartum depression [33] and another explored the experience of suicidality postpartum [34]. To date, no QIMS has considered the experiences of the birth environment for birthing mothers and the impact on maternal mental health. A synthesis of the literature qualitatively evaluating women’s perspectives on what is necessary to have a safe and sacred healing environment for mothers could bolster understanding of how hospitals could better support birthing mothers. As such, this study uses QIMS to answer the following research question: what is necessary to have a safe and sacred healing environment for mothers?

### Theoretical framework

This study sought to understand how birthing mothers experienced the birthing environment and which environmental factors contributed to a safe and sacred healing environment for mothers. As such, the Theory of Supportive Care Settings (TSCS) was used to frame this synthesis [35].

### Theory of supportive care settings

**Fig. 1:**
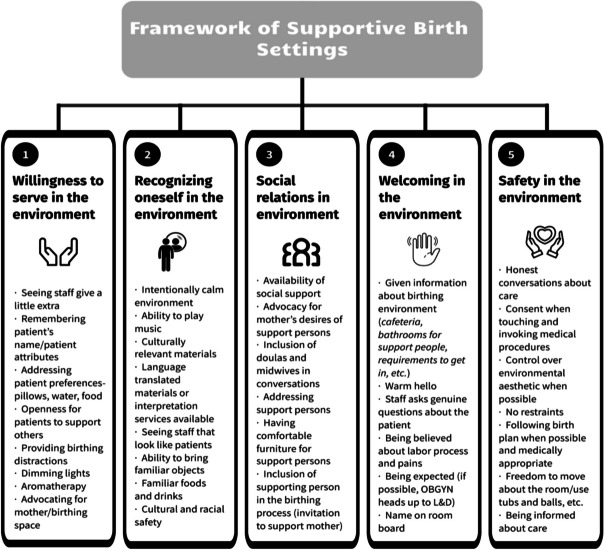
Framework of theory of supportive birth settings

Theory of Supportive Care Settings (TSCS) was created through research to have a theoretical understanding of which “processes supported a supportive care setting” [35]. TSCS was developed using three different care settings–a hospice, geriatric, and acute care ward, through qualitative interviews with patients, significant others, and care staff’s experiences. Although TSCS was not developed within the birthing environment, given the raise of childbirth induced P-PTSD, it is appropriate to apply the concepts to the birthing environment. One aspect of this synthesis is to assess the utility of the application of TSCS to the birthing environment using it as the main theoretical approach. There are five main processes the theory addresses as creating a supportive care environment: experiencing welcoming in the environment, recognizing oneself in the environment, creating and maintaining social relations in the environment, experiencing a willingness to serve in the environment, and experiencing safety in the environment. An applied theoretical framework was created (Fig. 1).

#### Experiencing welcoming in the environment

Experiencing welcoming in the environment has three properties which are intensely experienced when the patient first enters the healthcare setting [35]. *Being expected* is the first property that involves the care setting knowing the patient is coming. This happens by having the patient’s name displayed and knowing pertinent information about the person before the beginning of care [35]. *Being seen* entails a warm welcome upon entering the care setting, having personal introductions, and care staff showing an interest [35]. Lastly, *being invited* consists of being shown around the care setting for the patient to become familiar with the environment and the people within [35].

Certainly, experiencing welcoming in a care setting, such as a hospital, heightens mood among patients and increases their satisfaction with their experience of the care setting [36]. Within a birthing environment, there is also evidence that being believed and welcomed upon arrival to the hospital increases the satisfaction of mothers as well as enhances their birthing experience [37].

#### Recognizing oneself in the environment

Within TSCS, recognizing oneself in the environment encapsulates the intensity of which patients recognize themselves within the care environment [35]. For example, environments that are perceived as too sterile do not allow the patient to recognize themselves in the environment. Being able to recognize oneself in the care setting includes being in a familiar and calm environment [35]. A familiar environment includes objects that are familiar to the patients, as well as beauty in the environment that includes windows and warm colors [35]. Further, a calm environment has minimal loud noises from machines, phones, and patients are allowed to move freely [35]. Features of familiarity in the birthing environment can reduce the length of labor and reduce pain intensity [38].

#### Creating and maintaining social relations in the environment

Creating and maintaining social relations in the environment within TSCS describes the social relations a patient develops that create ease within the environment [35]. Within this concept, there are two processes: staying in contact with social relations and creating new social relations. Staying in contact entails the patient’s ability to stay in contact with those in their social circles while undergoing care and can include environmental factors that facilitate this such as access to a personal phone and privacy to visit with social relations while in care. Creating new social relations explains the way patients can create new social relationships through positive interactions such as those that include laughter and support from care staff or others in the care setting. The process further includes the structural environment and facilitation of such connections, including openness of concept, support places, and comfortable furniture in private and common areas of the care setting [35].

This process of TSCS is again supported in literature regarding birthing environments. Availability of social support is integral to the birthing experience and increased access to social support creates better birthing outcomes and perceptions of birth [39]. Similarly, those supporting the birth need to feel welcomed and included in the birth environment, and there are specific aspects of the built environment that facilitate increased support during birth such as familial alcoves in birthing rooms and increased attempts at including the supporter by care setting providers [40].

#### Experiencing a willingness to serve in the environment

The willingness to serve in the environment from TCSC involves both care staff and patients. In TSCS doing a little extra and receiving a little extra are the processes that promote a willingness to serve. To the patients, seeing the care staff demonstrate thoughtful actions shows the staff’s willingness to serve. These actions can include things like remembering a patient’s preferences for their pillow or water temperature or arranging food in an appealing way. The willingness to serve can also come from patients though; some patients reaching out to other patients to give support or even just showing caring attitudes towards either nurses or other patients. For patients, an environment which demonstrates the willingness to serve is one when care staff do things without being asked, are intuitive in their approaches, and do not make the patient feel like a burden [35].

Within the birthing environment, willingness to serve can look like staff providing welcome distractions from the birthing process through music or aromatherapy, dimming lights, changing ambient temperature, and ensuring loud sounds are minimal. Further, care staff can exhibit willingness to serve by advocating for the birthing mother to have less people in the room, creating a familiar space, and providing comfort [38].

#### Experiencing safety in the environment

TSCA defines safety in the birthing environment as the safe feelings that arise from knowing what is happening, feeling informed, being comforted, and feeling trustful of care providers. Understanding what is happening includes, knowing what is happening, having information in an accessible language, and being aware of the course of events. For the patient, being is safe hands means having trust in the providers through honest conversations, knowing that their needs and requests are honored, and that the physical environment is clean, organized, and aesthetically pleasing rather than chaotic and messy [35].

The safety in the birthing environment often ties honest conversations and knowing needs and requests will be met to feel in control over the birth and the experience. Feeling in control of the birth environment can also include creating a familiar, homey space by being allowed to personalize the space with music, design elements like personal photos, pillows, or plants, and controlling the temperature and lighting [40]. In addition, knowing that healthcare providers are respecting the birth plan as much as possible and supporting freedom to move and move through the birth process in their own way [38]. Furthermore, machinery that ties the mother down, inhibiting freedom to move, can be distracting and reduce the time midwives or nurses spend in the birthing room, diminishing the birthing mother’s trust in care providers [41].

Despite the lack of use of TCSC in birthing environment literature, all five concepts from TCSC are found within the existing literature to be recommended for use in birthing environments. That said, there is not a synthesis to date utilizing the framework to evaluate qualitative perspectives of the birthing environment. This review aims to organize the existing qualitative literature within TCSC to provide a roadmap for birthing space design that aligns with a supportive care environment, with the hopes of creating more functional birthing spaces which may reduce the rates of maternal mental health challenges following the birth of a child.

## Method

### Ethics, consent for publication, availability of data and materials

The data used in this study are derived from publicly available, published research articles and thus, in the public domain. Similarly, Institutional Review Board approval was not required since all data used were in the public domain in publicly available, published research articles. Informed consent was not required as no participants were recruited to participate in this study. There is no identifiable information of participants used in this method nor do we as consumers of previously published qualitative research have access to the original data.

### Design

QIMS is a method that lets researchers find a deeper understanding of a phenomenon or shared experience using qualitative journal articles as secondary data. QIMS is focused on researchers synthesizing previously published qualitative findings on a topic across the literature to reveal insights of participants’ experiences with a phenomenon [30]. This process includes creating a research question, conducting a systematic search of existing literature, and finally analyzing identified articles through theme extraction, synthesis, and triangulation [30].

QIMS has a set analysis process that involves reviewing the original authors’ published themes, as well as the participant's quotations in the manuscript. Themes and quotations are extracted and compiled into a new dataset to capture participants’ experiences of shared phenomenon across literature, providing a larger, more diverse sample size.

**Fig. 2:**
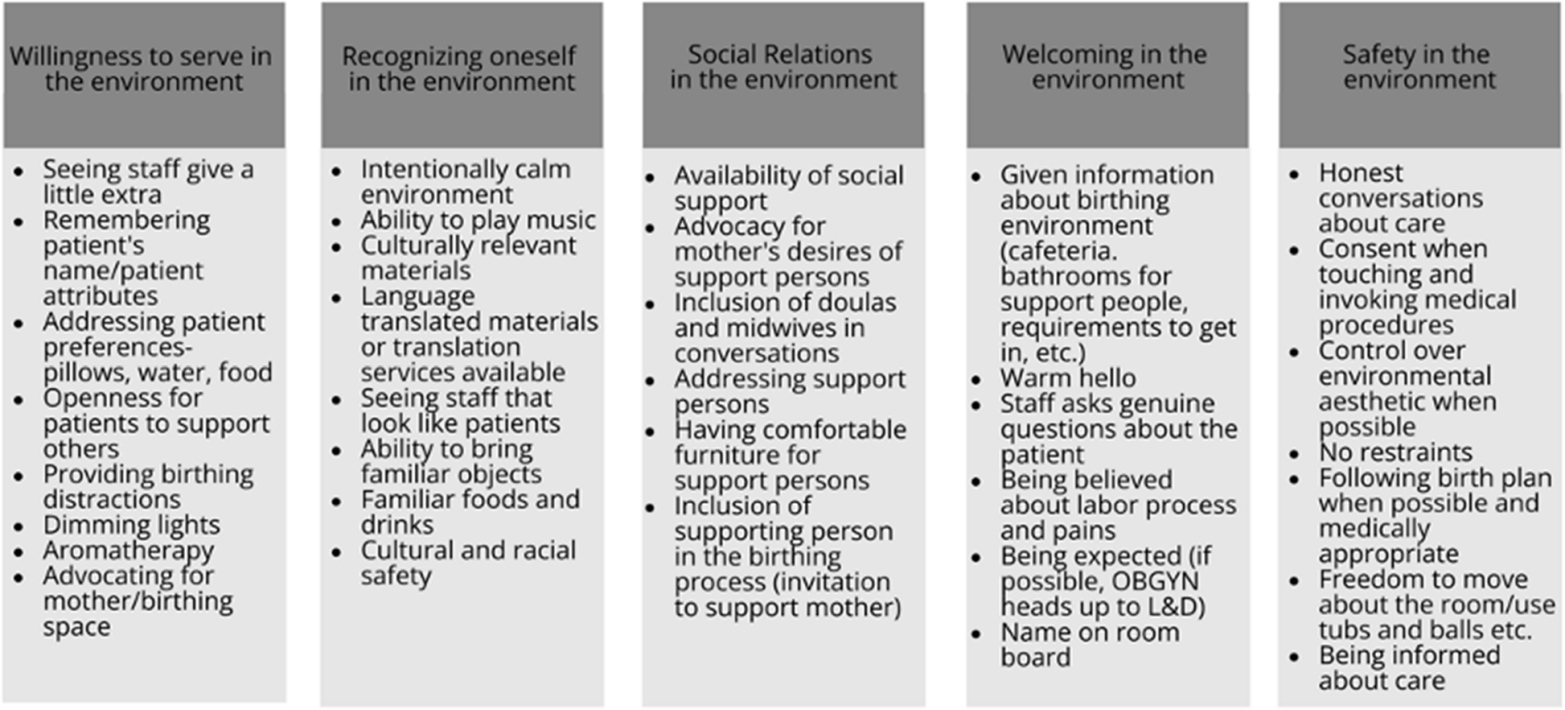
Associations of birthing environment to Theory of Supportive Care Settings

Sometimes, the analysis ends with a methodological reduction as well. Methodological reduction is an accepted method within phenomenological inquiry that permits researchers to understand the phenomena being observed through a new contextual lens allowing for further abstraction [42]. That said, due to the paucity of research evaluating what is necessary to have a safe and sacred healing environment for mothers, this study utilized a rare approach to QIMS wherein the theoretical framework was provided at the outset of the study to guide the entirety of the synthesis. This deviates from the more inductive approach of traditional QIMS, but this deductive approach allows for a more pointed answer to a specific research question that seeks to operationalize a construct within a distinctive context or population and has been used previously [30]. Essentially, this analysis approach used a combination of both QIMS and theory-testing deductive analysis methods. The theory guides each step of the QIMS process, and specific steps have been applied (see Fig. 2). This combined approach is formalized here and is called QIMS-DTT [deductive theory testing].

First, in line with theory-testing deductive analysis [43], a qualitative question was posed with a specific theoretical lens in mind, in this case, Edvardsson’s Theory of Supportive Care Setting. Then, following QIMS, a systematic search of the literature was conducted using PRISMA guidelines [44]. The keywords for the initial search included “birth or childbirth or labour or labor or delivery or birthing” as subject terms. The key terms “experiences or experience” and “qualitative” were added to “in abstract” as well as “birthing experiences” and “birthing perceptions.” Key terms were searched within the following databases: ERIC, Academic Search Complete, APA PsycInfo, CINAHL Complete, Family Studies Abstracts, MedicLatina, MEDLINE, Psychology and Behavioral Sciences Collection, Alt HealthWatch. This initial search yielded 5,688 articles. After duplicates were removed 5,167 articles remained. The title and abstract screened for content relating to the desired topic, and inclusion and exclusion criteria were applied.

Inclusion criteria were that the studies were U.S. based only, included pregnant women’s experiences of hospital or birthing center birth, and were qualitative research with quotations presented in the article. Inclusion was limited to U.S. based studies given that birthing practices differ vastly across the world; focusing on the U.S. provides homogeneity of context for understanding the birthing environment impact. Furthermore, even though the U.S. is a high resource country, the perinatal care system is considered unique as requires private pay insurance and not every woman has access to Medicaid or Medicare federal and state funded health insurance programs [45]. Furthermore, among 11 high resourced countries, the U.S. has the highest maternal mortality rate, which some scholars attribute to how the U.S. has the lowest supply of obstetricians and heavily lacks midwives and insurance coverage for midwifery care [46]. Theory was incorporated here as well as an inclusion criterion, and the results were filtered through the operationalization of Edvardsson’s Theory of Supportive Care Setting used for this study. Using the five constructs of the theory that were operationalized for this study, the articles were included if authors discussed at least one construct from the theory (the constructs that articles discussed can be found in Table 1). Articles not discussing at least one of the five constructs of the theory were excluded. In addition, other exclusion criteria included articles discussing future births or expectations about future births, choice of location for birth, mode of delivery, labor pain, healthcare providers’ perspectives, existing reviews or syntheses, and articles discussing techniques of or towards birthing [e.g., acupuncture, Lamaze, education]. After title and abstract screening, 3,178 articles were excluded, leaving 43 articles to be screened full text. During the full-text screen, 12 articles were excluded, leaving 31 total articles to be included in the QIMS.

**Table 1:** Components of Edvardsson’s theory of supportive care settings

Categories	Experiencing welcoming in the environment	Recognizing oneself in the environment	Creating and maintaining social relations in the environment	Experiencing willingness to serve in the environment	Experiencing safety in the environment
Properties	Being expected	Being in a familiar environment	Staying in contact	Doing a little extra	Understanding what happens
	Being seen	Being calm in the environment	Making new contacts	Receiving a little extra	Being in safe hands
	Being invited				
Articles	Experiencing welcoming in the environment	Recognizing oneself in the environment	Creating and maintaining social relations in the environment	Experiencing willingness to serve in the environment	Experiencing safety in the environment
Attanasio, McPherson, & Kozhimannil, 2014 [47]		X	X		X
Beebe & Humphreys, 2006 [48]	X	X	X	x	
Bernhard et al., 2014 [49]	X	X	X		X
Boucher et al., 2009 [50]		X	X		X
Brooks et al., 2016 [51]		X	X		X
Fair & Morrison, 2011 [52]	X	X	X		X
Finn, 1994 [53]		X	X	x	
Fowles, 1998 [54]		X	X		X
Gardner et al., 2016 [55]	X	X			
Hall et al., 2018 [56]		X	X	x	X
Hill, Hunt, & Hyrkäs, 2012 [57]		X	X		
Lipson & Rogers, 2000 [58]		X	X		X
Low et al., 2003 [59]	X	X	X		X
Low & Moffat, 2006 [60]	X	X		x	
Lynch et al., 2019 [61]	X	X	X		X
Lyndon et al., 2018 [62]	X		X		X
Matthews??				x	
McKinney, 2006 [63]		X	X		
Qureshi & Pacquiao, 2013 [64]		X	X		
Raines & Morgan, 2000 [65]	X		X		X
Sauls, 2010 [66]		X	X		X
Seo, Kim, & Dickerson, 2014 [67]	X	X	X		
Sheffield & Liddell, 2023 [68]		X			X
Smeltzer et al., 2017 [69]		X	X		X
Taniguchi & Baruffi, 2007 [70]		X	X		X
Tiedje & Price, 2008 [71]		X	X		X
VandeVusse, 1999 [72]		X	X	X	
Yeo & Maeda, 2000 [73]	X	X	X		

### Analysis

Following this approach (inclusive of both QIMS and theory-testing deductive analysis) we have formalized within this study, the original themes (Table 2) from the articles were organized by one researcher into appropriate theoretical assumptions that most aligned with the constructs of TSCS (See Table 1–providing theoretical triangulation). Then, the quotations from each article were extracted and uploaded to qualitative software, atlas.ti (v.8.1). The quotations were coded deductively by the first two authors using the theoretical framework as a guide for thematic development. The themes were then aligned with each of the five theoretical constructs by unanimous rating. This process provided a layer of analyst triangulation additional to the triangulation inherent in QIMS design resulting from triangulation in the individual studies prior to the QIMS.

**Table 2:** Original themes

Author(s), Year	Original Published Theme
Attanasio, McPherson, & Kozhimannil, 2014 [45]	1. Individual-level maternal factors a. Previous birth experience b. Plans and interactions 2. Clinical encounter and health systems factors a. Procedures b. Complications or unforeseen factors 3. Role of providers 4. Physical environment 5. Logistics of coordination with providers and space
Beebe & Humphreys, 2006 [48]	1. Expectations 2. Identifying labor – “The real thing’ 3. Managing symptoms and emotional responses 4. Supportive resources 5. Decision making about hospitalization – “Going in”
Bernhard et al., 2014 [49]	1. Choices and Empowerment 2. Interventions and Interruptions 3. Disrespect and Dismissal 4. Birth Space 5. Connection
Boucher et al., 2009 [50]	1. Safety and Better outcomes 2. Intervention-Free 3. Negative Previous Hospital Experience 4. Control 5. Comfortable Environment
Brooks et al., 2016 [51]	1. Lumbee Mothers’ Descriptions of Having a Premature Infant in the NICU a. Premature Birth and NICU Experience i. Relationship with Providers ii. Maternal Role Alteration 2. Lumbee Mothers’ descriptions of parenting a premature infant a. Infant health and development b. Posttraumatic stress symptoms 3. The influence of Lumbee culture on parenting a premature infant a. Balancing traditional and nontraditional medicine b. Pride in the Lumbee heritage
Fair & Morrison, 2011 [52]	1. Preparation a. Knowledge/planning b. Social support 2. Communication a. Woman-initiated i. Communication of needs ii. Asking questions b. Provider-initiated i. Providing information ii. Shared decision making 3. Support a. Reassurance b. Encouragement c. Physical assistance d. Support of mother-infant relationship 4. Respect for wishes
Finn, 1994 [53]	1. Universal culture care patterns a. Culture care accommodation: A professional nurse care mode 2. Euro-American cultural values and childbirth 3. Encouragement: A discovered care construct
Fowles, 1998 [54]	1. Positive experiences 2. Frustrations a. Lack of control b. Lack of knowledge c. Negative perceptions of health caregivers d. Relationships among the subcategories of frustrations
Gardner et al., 2016 [55]	1. Prenatal period a. Processing sensations 2. Intrapartum period a. Processing sensations b. Needing to have 3. Postpartum period a. Walking in the dark b. Motherhood on my own terms c. New sensory experiences
Hall et al., 2018 [56]	1. The essence of childbirth: Keeping it together or falling apart 2. The characteristics of keeping it together and falling apart a. Confidence: Believing in the power of oneself b. Comfort: The power to make the self feel better c. Agency: The power to get what you need d. Connection: The power to choose trustworthy supporters
Hill, Hunt, & Hyrkäs, 2012 [57]	1. Pregnancy as a natural experience for women a. Part of Somali life b. Role of faith c. Scientific basis for Western medicine 2. Value and relevance of prenatal care a. Unfamiliarity with purpose b. Relevance of medical care c. Advanced technology 3. Lack of control and familiarity with delivery in the United States a. Home delivery versus hospital care b. God has control c. Prevention of tearing 4. Balancing the desire to breastfeed with practical concerns and barriers a. Feeding the baby b. Optimal duration 5. Discomfort with mental health issues a. Stigma b. Depression after delivery c. Use of psychotropic medications 6. Challenges in the health care system a. Experiencing access to health care b. Getting to appointments and differing perception of time c. Role of many providers
Lipson & Rogers, 2000 [58]	The women’s perspective The effect of the disability Women’s resources Personality and approach The health care system a. Pregnancy b. Birth experiences c. The postpartum period/infant care
Low et al., 2003 [59]	Planning for the birth experience Natural childbirth Greater focus on the baby Media as source of information Pain Adolescents’ relationships with health care providers
Low & Moffat, 2006 [60]	“Don’t trust your body, trust us” This is “not right” This too is labor!
Lynch et al., 2019 [61]	Time preceding admission: Feeling dismissed Transfer or admission to a tertiary care center: Anxiety and doubt The birth: fear of the outcome Postpartum: Reflection and communication
Lyndon et al., 2018 [62]	Safety experienced as a continuum Environment and organizational factors Interpersonal interactions The power of human connection
McKinney, 2006 [63]	Husbands and partners as the ultimate supporters Having a “natural” birth without fear Relaxation and preparation as roads to empowerment Teachers, methods, and materials Relationships with medical providers and caregivers
Qureshi & Pacquiao, 2013 [64]	Significance of collective support in early pregnancy Contrasting perception of support from in-laws and own family Lack of familiarity with the U.S. health care system and financing of health care services Contrasting cultural expectations, beliefs, and practices in Pakistan and the United States Adaptive strategies to changes in cultural practices Emergent changes in patterns of decision making and gendered roles
Raines & Morgan, 2000 [65]	What made you feel comfortable? Who should be present? What is the significance of that person being with you? How would you like your family to be involved?
Sauls, 2010 [66]	Respectful nurse caring Assistance with pain control Nurse support of adolescent’s support person Childbirth guidance
Smeltzer et al., 2017 [69]	Themes related to labor and birth experience Preference for type of delivery Clinicians and some women expected no labor pain Fears prompting active advocacy Positive experiences Themes related to obstetrical anesthesia a. Importance of consultation with the anesthesia team b. Decisions about epidural/spinal vs. general anesthesia c. Failed epidural with repeated efforts d. Fear of injury related to anesthesia
Seo, Kim, & Dickerson, 2014 [67]	1. Feeling lost in the healthcare environment 1. Language as a barrier 1. Facing unfamiliar health care system 1. Being socially isolated 2. Having limited choices in physician or hospital a. Relying on physicians for decision making 3. Holding to Korean tradition a. Expecting cultural sensitivity b. Practicing Korean traditions with some modification 4. Seeking information and support a. Bridging the gap
Taniguchi & Baruffi, 2007 [70]	Difficulties during pregnancy or after childbirth Mental health during pregnancy and postpartum Coping with difficulties
Tiedje & Price, 2008 [71]	Trust Control Information
VandeVusse, 1999 [72]	Contested control: Unilateral decision making Contested control: Through refusal Contested control: Through adaptation Unilateral and uncontested control: Through agreement Suspended control while waiting: Through no active decision Shared control: Joint decision making Shared control: Through explanations Shared control: Through requests
Yeo et al., 2000 [73]	Negative factors Language barrier Ultrasonography Prenatal vitamin supplementation Episiotomy Positive factors Epidural analgesia Caregiver-client relationship

### Instrumentation

In addition to the analysis process, it is also important for researchers to bracket, or disclose, their experiences with a phenomenon to increase the trustworthiness of the synthesis. The authors are the main instruments of this study, as is frequently the case in qualitative research. To further lend credibility and transparency to the QIMS process, brief descriptions of the authors can be found in Table 3. The authors purposefully include two mothers–one who experienced Postpartum Mood and Anxiety Disorders (PMADs) and one who did not, and two women who were not mothers at the time of this writing. This intentionally focused toward balancing any biases the two mothers might have brought to the analyses given their experiences further explained in Table 3.

**Table 3:** Author positionality statements

Author #	Positionality Statement
1	The first author specializes in maternal mental health, perinatal care environments and trauma-informed perinatal care, and culturally responsive perinatal care. She is mother who did not experience maternal mental health challenges, however, has worked closely with Indigenous communities of women who have had PMADs
2	The second author
3	The third author is a doctoral student. She is focused on researching sex education for Latinas to promote sexual safety and decrease sexual victimization. Her hope is to offer culturally sensitive sex ed to increase maternal-infant bonding in the lifespan
4	The fourth author is one of the original co-creators of the QIMS method and originally piloted this revised approach to QIMS. She is also a mother of two sons and experienced postpartum depression after the birth of her first son. Her primary research focus is suicide and she has explored suicidality among postpartum mothers as a subfocus

### Sample

The final sample included 30 qualitative studies giving ear to the voices of 1,802 postpartum mothers. These mothers ranged in age from 12 to 71 and represented a wide range of races and ethnicities. For more demographic information including data collection methods and settings, see Table 4.

**Table 4:** Summary of Included Articles

Authors, Year	Methodology	Aim	Sample	Age, Race/Ethnicity	Study Location
Attanasio, McPherson, & Kozhimannil, 2014 [47]	Mixed methods, survey with open-ended questions;	Explored qualitative themes of positive birth experiences	519 Nulliparous mothers and 1,054 parous mothers	Nulliparous: 38.8& 18–24 years old, 27.3% 25–29 years old, 23.2% 30–34 years old, 10.6 35 + years old; 62.4% White, 11.9 Black/African American, 21.5% Hispanic, 4.2% other; Parous: 22.2% 18–24 years old, 27.5 25–29 years old, 26.4% 30–34 years old, 24.0% 35 + years old; 62.3% White, 12.3% Black/African American, 20.6% Hispanic, 4.8% other	United States
Beebe & Humphreys, 2006 [48]	Ethnography, interviews	Understanding experiences of admission to hospital	23 first time mothers	Ages 7 18–25 years old, 6 26–30 years old, 7 31–35 years old, 3 36–40 years old; 20 Caucasian, 2 Hispanic, 1 other	West Coast
Bernhard et al., 2014 [49]	Qualitiative descriptive design, focus groups	Understanding the reasons for avoiding hospital birth after experiencing hospital birth	20 women	Ages 22- 42 years old, 19 self-identified as white, 1 multiracial	West Michigan, United States
Bourcher et al., 2009 [50]	Qualitative descriptive design	Understanding the reasons for avoiding hospital birth after experiencing hospital birth	160 women who planned to give home births	Ages 20–65 years old, Mean age of 35, 2 Asian, 9 Hispanic, 139 White, 39 Not specified	United States
Brooks et al., 2016 [51]	Longitudinal, descriptive, qualitative study	To understand American Indian mother’s perceptions of hospitalization and birth	17 Lumbee mothers and their premature infants	Ages 17–42 years old, Lumbee	South Eastern North Carolina, United States
Fair & Morrison, 2011 [52]	Semi-structured interview	To explore women’s perceptions of practice that facilitate control during birth	31 mothers who were 6 weeks postpartum	Ages 18–35; 13 African American, 14 Caucasian, 4 other	Southeastern United States
Finn, 1994 [53]	Phenomenology, interviews	To understand meanings of care/non-care experienced during birth	10 mothers; 9 nurses who cared for them	Ages 21–33; all Euro-American	Midwestern United States
Fowles, 1998 [54]	Questionnaire	To understand discrepancies between expectations and actual birth	77 mothers	Ages 18–35; race/ethnicity not reported	Midwest United States
Gardner et al., 2016 [55]	Qualitative survey	To understand the childbearing experiences of women with Asperger's Syndrome	8 women with Asperger syndrome	Ages 27–52; race/ethnicity not reported	United States
Hall et al., 2018 [56]	Phenomenology, semi-structured interviews	To explore the complexity of women’s birth experiences	8 mothers who had uncomplicated birth in home or hospital	Ages 25–39; 4 Caucasian, 2 African American, 2 Hispanic	Atlanta, Georgia
Hill, Hunt, & Hyrkäs, 2012 [57]	Focus groups	To explore Somali immigrant women’s experiences of healthcare during pregnancy and birth	18 pregnant and postpartum Somali women who had given birth in the U.S	Ages 27–42; all Somali	Northeastern United States
Lipson & Rogers, 2000 [58]	Semi-structured interviews	To explore pregnancy, birth, and postpartum experiences of women with mobility-limiting physical disabilities	12 mothers with mobility-limiting physical disabilities	Not reported	United States
LoGiudice & Beck, 2016 [74]	Phenomenology, one-on-one interviews	To understand experiences of pregnancy and birth for survivors of sexual abuse	8 survivors of sexual abuse	Ages 38–58; 7 Caucasian, 1 Hispanic	Connecticut
Low et al., 2003 [59]	Extended case methodology	To understand meaning of childbirth experiences for adolescent mothers	25 adolescents	Ages 13–18; 10 Euro American; 3 African American, 4 Mexican American, 1 Asian	Southeastern state
Low & Moffat, 2006 [60]	Semi-structured interviews	To explore women’s perceptions of transitioning to birth facilities while in labor	24 first time mothers within 1 to 3 weeks of giving birth in hospital	Age not reported; all Caucasian	Midwestern United States
Lynch et al., 2019 [61]	Semi-structured interviews	To understand the experiences of periviable birth	10 mothers 24 h to 12 days postpartum	Ages 22–35; 60% White, 10% Hispanic, 30% unknown	United States
Lyndon et al., 2018 [62]	Semi-structured interviews and groups	To understand women’s birth experiences during hospital birth	17 mothers	Ages 29–47; 13 Caucasian, 1 Asian, 3 unknown; 2 unreported	San Francisco, California
Mackey, 1988 [75]	Semi-structured interviews	To understand how women describe and evaluate their labor and delivery experience	60 mothers from Lamaze classes	Ages 21–37; 85% Caucasian, 6.5% African American, 6.5% Latin American, 2% Asian American	Columbia, South Carolina
Matthews & Callister, 2004 [76]	Semi-structured interview	To understand the perceptions of maintenance of dignity while laboring and giving birth	20 primiparas women	Mean age 23, all white	Western United States
McKinney, 2006 [63]	Online qualitative questionnaire	To understand the experiences of birth for those who used the Bradley Method	15 mothers who used the Bradley Method	Not reported	United States
Qureshi & Pacquiao, 2013 [64]	Interviews	To understand the birth experiences of Pakistani women immigrants giving birth in the U.S	26 Pakistani mothers who gave birth in U.S	Average age 38.5; all Pakistani	New Jersey
Raines & Morgan, 2000 [65]	Semi-structured interviews	The understand the meanings of “Comfort,” “presence,” and “involvement” in the context of the childbirth experience	20 mothers who had given birth within 72 h	Average age 26.3; 10 Caucasian; 10 African American	United States
Sauls, 2010 [66]	Quantitative survey with 2 open-ended qualitative questions	To identify labor support needs for adolescents to promote a positive childbirth experience	185 adolescents who had given birth within 48 h	15 12–14 years old, 64 15–16 years old, 106 17–19 years old; 17 White, 129 Hispanic, 36 African American, 3 mixed	Texas
Sheffield & Liddell, 2023 [68]	Semi-structured interviews from 18 to 71 years old (M = 51.71 years). Most participants (87.1%) reported having either a GED or high school degree. About half of the par- ticipants (51.61%) had some amount of postsecondary training or education. A large majority (93.54%) were covered by some type of health insurance, and most (83.87%) had at least one child. The average participant first gave birth at about 20 years old and, at the time of their interview, had two or three children	To understand better American Indian birthing people’s preferences for birthing type and place	31 female-identifying people; 83.87% of whom had given birth	18 to 71 years old; self-identifying as members of a state-recognized Indigenous tribe	Gulf Coast region of the United States
Seo, Kim, Dickerson, 2014 [67]	Hermeneutic phenomenology,	To understand Korean immigrant women’s experiences using healthcare services in the U.S. during childbirth	15 Korean immigrant women	Ages 29–42; all Korean immigrants	All across United States
Smeltzer et al., 2017 [69]	Semi-structured interviews	To examine the experiences of labor and delivery among women with significant mobility disabilities	22 mothers with a physical disability at the time of birth	Average age 34.8; 20 White, 2 Hispanic	United States
Taniguchi & Baruffi, 2007 [70]	Semi-structured interview	To investigate types of stress women experience giving birth in a foreign country	45 mothers born in Japan who gave birth in U.S	Age 21–46; 24 Japanese, 10 Caucasian, 11 other	Hawaii
Tiedje & Price, 2008 [71]	Focus groups	To examine women’s experiences, attitudes, and opinions about childbirth	12 mother who were 2 to 4 months postpartum	Ages 23–37; all White	Midwestern United States
VandeVusse, 1999 [72]	Secondary qualitative analysis	To clarify how decisions are made during labor	15 mothers	Ages 18–39; 12 Euro-American, 3 women of color	Midwestern United States
Yeo & Maeda, 2000 [73]	In-depth interviews	To examine Japanese couple’s perceptions and experiences of childbirth in the U.S	11 pregnant Japanese couples who gave birth in the U.S	Average age of husbands 35.2, average age of wives 32.9; all Japanese	Michigan

## Results

Using a theory-testing deductive analysis process in conjunction with QIMS, the analysis results in five themes with various subthemes. The supporting quotations can be found in Table 5. In addition, thematic constructs of TSCS were found across the included articles and the theoretical deduction was sound. Evidence of theoretical constructs can be found in Table 1.

**Table 5:** Supporting Quotations

Primary Theme	Subtheme	Supporting Quotations
**Theme 1: Service in the Environment**		So to come here and have people say, ‘This is what we can do, this is what we can’t do, these are your options’ and to have people actually sit [with you], and not only that, but seem like they care, because I didn’t feel like they care, because I didn’t feel like they cared in the other place. They [tertiary care providers] cared about what was going to happen. So that make things easier… its horrible. But it has been made easier by people that actually cared. (Brooks et al., 2016, Pg. 427)
		I just really loved the nurse. She was really upbeat and gave me a lot of compliments, like, ‘Oh, you’re doing a great job’ and ‘you’re so strong’. She was really supportive. (Matthews et al., 2003 Pg. 500)
**Theme 2: Recognizing Oneself within the Birthing Space**		
	***Subtheme 2A: Acknowledging Personhood***	I said, ‘Why, I can take the cords with me,’ and they wouldn’t let me… I had to use a bedpan. And that upset me. Then [a few hours later], I had to have a bowel movement. She said, ‘Okay, fine, you can go. I’ll get the bedpan.’ I said, ‘No, you have to let me get up.’ And they said, ‘No, we can’t let you.’ I tried to explain that I knew I was going to have a bowel member. Please let me go. No. And so after, not being able to hold it and not being able to argue any longer, I said, ‘Okay.’ They put the bedpan in. I had to have a bowel movement, so I had to sit up; meantime, the IV is getting all messed up, the blood is coming through the tubes… I’m crying. ‘Just let me go to the bathroom.’ No, they wouldn’t let me, because, I don’t know, they felt the baby’s heartbeat was getting, going down or something. I don’t know why. I succeeded in getting it out in total embarrassment. And I thought if I moved wrong it’s going to go all over the bed. And she [the nurse] said, ‘Don’t worry, don’t worry.’ And she had to wipe me. I cried. ‘This wouldn’t have happened if you had just let me go to the bathroom on my own.’ So she [the nurse] go everything under control. (VandeVusse, 1999, p. 46)
		I’m naked and everything was hanging out and they’re talking about strategy for changing the bed… and I’m not even a modest person, but that moment when I felt so horrible, just so bad, to spend so much time [doing that while I’m naked], I’ll never forget that (Lyndon et al., 2018, pg. 329)
	***Subtheme 2B: Validating Maternal Role***	It went too fast, but also I was not pleased with how they handled the situation after he was born. They took him from me and didn’t let me know how he was or what was wrong. They finally told my mother 2 ½ hours after he was born what was going on. Then they said that he was fine and that there was nothing to worry about, yet it took another 1 ½ hours before I got to see him. I feel that they should let mothers know what’s happening. (Fowles, 1998, pg. 238)
		No it would have been natural, because my sister had delivered her daughter in January. My son was delivered in March and I remember her having a [c] section and I remember her not knowing, for like hours after if she had a boy or a girl cause she kept waking up and asking what I had, is it, how is she, and she fall asleep and she's wake back up and say, so what’s going on? Is she okay? She couldn’t see her baby, couldn’t hold it.Soon as my child was born, I was able to hold and she was not able to, so no. I knew at that point that I was going to have a natural birth.” (Sheffiled & Liddell, 2023, pg. 28)
**Theme 3: Creating Connections with Support Systems**		
	***Subtheme 3A: Communication is Key***	I kept asking the nurse what’s happening. Asking questions and feeling comfortable with the answers, making sure I understood [helped].( Matthews et al., 2003 Pg. 23)
		The midwives… always asked before doing stuff, and generally kept their hands off. (Matthews et al., 2003, Pg. 24)
		I felt frustrated being flat on my back. The contractions were a lot harder and stronger when I was laying down that when I was sitting up. I didn’t have the freedom to sit up. I wish I’d asked more questions. I’d ask them to explain to me, ‘Why are you doing this? What is going on here? (Matthews & Callister, 2004, p. 502)
	***Subtheme 3B: Team Effort Among Providers***	They left you with your own labor and delivery and then the time that you needed the support system, they were there and took over. It was like everybody played their role in terms of dominant and subordinate when it was right to do that” (Mackey, 1988, pg. 24)
		There is a difference between being delivered and giving birth. I was giving birth and they [the nurse midwife and nurse] were assisting me, rather than me just laying there and being delivered. It was a team effort. There is a difference, and I was pleased. That’s the way I wanted it” (Matthews & Callister, 2004, pg. 502)
		When they push that button, everyone runs. So there were probably 15 people in the room… no one was really explaining. I was just listening for instructions… for what to do among all the chaos… it’s stressful… it was kind of like mass chaos” (Lyndon et al., 2018, pg. 327)
	***Subtheme 3C: Respect Forges Social Connection***	I had pain medicine for my oldest daughter. And the reason I had it was because I didn’t know any better. I didn’t know what was labor pain or anything. So they gave me one of those, “twilight” they call it, it’s a shot that they give you, and I was out. My second child, I was ten minutes in the hospital, he was born. So when I had my third one, I didn’t want nothing. Doctor says… “You got to have…” “Nope, I don’t want nothing.” So he put the thing [mask] on my face and, [said] “Take a deep breath,” I took a deep breath with my mouth closed and I had my baby natural. (Sheffield & Liddell, 2023, pg. 30)
		They respected the fact that I was in pain, that I had tears in my eyes and was about to cry. (Matthews et al. 2003 Pg. 501)
		[My obstetrician] knew… that I wasn’t talking out of fear… that I had some knowledge and education to support my decisions. She really believed I knew my body the best and was willing to help me… She listened and she read my chart and she said, ‘I see this is what you want… you and your baby to come out of this healthy and fine.’ … And she’s like ‘you have every right… (Smeltzer, Wint, Ecker, & Iezzoni, 2016, pg. 320)
		I mean, we had talked all this over before. And she agreed to everything that I had wanted. But then, at the time of the birth, actually things were a little bit different than I had authored. I was stuck in bed because of the monitor being there” (VandeVusse, 1999, p. 47)
**Theme 4: Being Welcomed into the Birthing Space**		
	***Subtheme 4A: Being Believed and Admitted***	The only thing I worried about was going to the hospital maybe too soon. You have that fear of getting there and… then having the doctor tell me that I could come in tomorrow, and kind of going over him and making that decision [to go in sooner], and worrying about it being wrong… I just thought it would be bad if we get there only to be told to go back home. It would be discouraging. (Beebe & Humphreys, 2006, p. 351)
		I had one contraction right after the other right there in the lobby and another one getting out of the elevator. It was really funny because the receptionist who was waiting there in the triage area sort of looked out and saw me there and thought, ‘Labor! We’re not sending her home.’ And I was thinking, you know, I hope they do not send me home cause, I don’t know, maybe it would be like failing somehow to show up at the hospital and think you’re in labor and you’re not. (Low & Moffat, 2006, p. 311)
	***Subtheme 4B: Comfortable Birthing Space***	We had the aromatherapy, we had CDs, we had tennis balls for massage…(Beebe & Humphreys, 2006, p. 349)
		… For me, it was very cold after the delivery and also during the delivery. I had chills a lot and had a troubled time after the delivery. The hospital was too cold (Seo et al., 2014, p. 312)
		I like the labor and delivery room at the hospital I was in… it was big and comfy and had room for anyone who wanted to be there and it had a cd player. (Attanasio et al., Pg. 1286)
**Theme 5: Feeling Safe Within the Birthing Environment**		
	***Subtheme 5A: Interpersonal Safety***	I had the same nurse stay with me the whole time I was in labor. It was really nice to have help from somebody I knew they whole time, and not have them keep switching on me (Matthews & Callister, 2004, pg. 501)
		I turned around and chose to be a woman doctor, and it must be because having the man obstetrician bothered me… I think I choose a female doctor and then a midwife around the fourth month because it felt more comfortable to me” (LoGuidice, 2016, pg. 478)
		I didn’t think it was a breeze, but the experience was so much nicer. It was a real experience… [This time] the doctor, my husband, myself, and the four nurses were there the whole time – so you always had a familiar face, which was nice” (Mackey, 1988, pg. 24)
		I was looking and there’s just so many people coming in the room and [the anesthesiologist said], ‘I don’t want you to look at them. I want you to look at me… I’m going to talk you through all this, and I just want you to focus on me.’ And so I did feel very safe. Even though I could hear other stuff happening, it was nice to like have that like okay, you’re it for me right now. Like this is all I have to focus on right now. So that was when I felt the safest.(Lyndon et al., 2018, Pg. 329)
	***Subtheme 5B: Confidence in the Healthcare Team***	I prefer to take advice from a doctor. He knows more than I do. I don’t want to make decisions that I don’t know much about” (Matthews & Callister, 2004, pg. 502)
		I wanted to try everything I could to save the baby. I knew there was more danger for me with a cesarean, but I didn’t care at all about that. What concerned me the most was not knowing what was going to happen with her? She wanted physicians to do, whatever you have to do, whatever was going to be the best outcome for her survival because as a parent, you always want to try. Pg. 427
		The third night, the night nurse is like… ‘What are you going? You’re starving your baby; this baby lost two pounds today.’ I thought, ‘Oh my God, how can that be? I’ve been breastfeeding and all the stuff…’ She said, ‘I have to give him a bottle.’ We were told, ‘If you give him a bottle, then he’s not going to want to do the breast.’ The nurses were good, except that one night nurse, because he didn’t lose two pounds, he lost two ounces. I didn’t know; I had to trust her” (Lipson & Rogers, 2000, pg. 22)
	***Subtheme 5C: Feeling in Control of the Birth***	I felt like they [medical staff] were trying to involve me in making decisions and I had control in that way. But I know I couldn’t do this by myself. I was at the hospital because I needed professionals. I felt in control but I also felt very dependent. (Matthews & Callister, pg. 502)
		There were two obstetricians, one older, one very young and "Doogie Houser" looking. So we went in to talk to the obstetricians about our options, our birth plan in hand, still filled with naive optimism about how this birth will go. The older obstetrician said. "No way, you're too short for natural childbirth (?!?!?) [emphasis by participant]." He also insists on an X-ray to see if my pelvis is, "adequate." I flatly refuse. "Well," he says, "let's schedule a c-section," The younger obstetrician sighed and said, "Well, I'm willing to let you TRY [vaginal birth], but no squatting position, no dim lights, no new-age, whale-song mood music, and it would have to take place in the OR. prepped and ready for a csection. Oh, and you'll need an episiotomy. (Mckinney et al., 2006, Pg. 28)
		I’m the one who’s in charge of the childbirth experience, not the doctors, or the nurses, or the hospital. This is between me, the baby, and my husband. Everybody else is there to support, not to take control. (Matthews et al., 2003 Pg. 502)

### Theme 1: service in the environment

The first theme consists of ways that participants experienced service within the birthing environment. This service can be either from the healthcare team or the woman themselves and can be expressed in ways more encompassing than just direct labor. Participants described providers who exhibited exceptional care as a memorable part of their birthing experience. This aspect of service within the environment contributed to warm feelings towards their providers and allowed them to feel important and cared for. Many described how taking time out of their busy schedules to focus on the woman one-on-one, accommodating disabilities or medical conditions without being asked, and going out of their way to encourage and empower women was how a provider demonstrated “above and beyond” care.

### Theme 2: recognizing oneself within the birthing space

The second theme described how birthing persons saw themselves within the birthing space. This included their personhood being acknowledged and their maternal role being validated by providers.

#### Subtheme 2A: acknowledging personhood

Recognizing oneself within the environment should be facilitated by feeling acknowledged as persons with dignity. For participants in these studies, this was expressed in their experiences of not having their personhood acknowledged and valued during the birthing process. One participant was not allowed to walk to the bathroom and was also not clearly told why. Her dignity was wounded, and the situation introduced emotional trauma into her birth story. Other women had a similarly emotionally traumatic experience that compromised their dignity and devalued their personhood.

#### Subtheme 2B: validating maternal role

Validation in becoming a mother is an important step in a woman’s transition into motherhood. The birth is an experience that will forever impact how the person views their maternal role. Many participants felt that their role as mother was overlooked by providers or not validated in a way that made them feel unequipped to mother their children. Often, participants described how providers made decisions for their newborns for them without consulting or trusting them to make such decisions.

### Theme 3: creating connections with support systems

The third theme describes the ability of participants to forge or maintain social connection while experiencing birth. This could be availability of social support through communication from providers or through inclusion of support persons. Furthermore, disrespect hampered the formation of social connections.

#### Subtheme 3A: communication is key

This subtheme revolved around the necessity of communication to forge a strong, trusting social connection between provider and women. This communication included informing the women of medically necessary interventions and allowing them to understand the necessity of them before consenting when medically possible. Communication also included introducing themselves and accepting a patient introduction genuinely through learning womens’ names and making eye contact and gathering consent before touching the client. When providers communicated in this fashion, the participants indicated that they felt a stronger social bond to the providers and their trust and satisfaction with them was increased.

#### Subtheme 3B: team effort among providers

Relationships required a team effort, which meant that multiple providers needed to be on the same page and operating in good communication with one another to support mothers. Participants in the included studies described how both providers and the birthing person, as well as their support people could work together to ensure the birthing process was a positive one. Others explained that when providers did not work together or communicate among each other the birthing process felt chaotic and disjointed, leaving them feeling unsatisfied and unsafe.

#### Subtheme 3C: respect forges social connection

This sub theme describes how care providers can forge social connection with their patients through respecting the wishes of the birthing person. Examples included respecting their birthing plan even when it was not medically necessary, allowing the birthing person to make choices about pain interventions, and not respecting the minimal birthing requests that were not related to medical interventions. Conversely, not hearing or respecting the birthing person created a negative experience which was detrimental to social connection in the birthing space.

### Theme 4: being welcomed into the birthing space

The fourth theme that emerged encompassed participants’ desires to be welcomed into the birthing space. This involved experiences of being admitted into the maternity ward or birthing suite upon arrival at the hospital and being made to feel comfortable in the space.

#### Subtheme 4A: being believed and admitted

Participants within the included articles discussed the importance of being believe when they presented to the hospital in what they perceived as active labor. Participants described being unsure if the sensations they were feeling were in labor and expressed anxiety as to whether they would be admitted into the maternity ward. Participants worried that if they arrived at the hospital too early, they would be treated poorly for “over-reacting” and be sent home, even though they were in pain. Participants also described the feeling of being rejected as failure. Being admitted into the birthing space was crucial for participants in the included articles to feel supported and validated.

#### Subtheme 4B: comfortable birthing space

In addition to being admitted, having the birthing space be comfortable was also necessary for participants to feel welcomed. Participants described spaces that had enough room for all their family members, single-occupancy rooms that allowed the birthing mother to have the whole room to herself, and rooms that had calming items present to be the most comfortable. In addition, participants in the included articles described experiences of uncomfortable spaces. Several participants expressed discomfort at having to be moved to multiple locations within the hospital. Participants also found hospitals challenging to navigate which caused stress on the family and the laboring mothering. Some participants described how the temperature of the space affected them as well, with the ability to control the temperature helping them to feel comfortable, both themselves and their families.

### Theme 5: feeling safe within the birthing environment

The fifth theme encompasses various ways birthing persons felt or did not feel safety in the birthing environment. Either through consent in procedures, being able to follow birth plans, having freedom to move, and having trust and confidence in the healthcare team, there were many ways participants expressed their perceptions of safety in the birthing environment.

#### Subtheme 5A: interpersonal safety

This theme described how interpersonal relationships contributed to feeling safety in the birthing environment. Participants in the original studies talked about how they took action to ensure they had interpersonal safety through choosing obstetricians that felt safe to them, either due to gender or validation tactics. Others described how having continuity of care when possible created safe feeling interpersonal relationships, such as having the same nurse throughout or when they did change shifts- the outgoing nurse took extra steps to introduce the new nurse and supported the forging of an interpersonal relationship between birthing person and new nurse. Having a familiar face consistently throughout the birthing process was comforting. In addition, many quotations described how a provider could focus on the woman in a way that was comforting and forged and interpersonal connection by ensuring they knew they were being heard and supported.

#### Subtheme 5B: Confidence in the healthcare team

Feeling safe in the birthing environment was also influenced by how much confidence the women had in their healthcare team. Some participants in the original study described how they trust doctors because they know better through education, while others felt like their care providers were not listening to their concerns, eroding their trust and making them feel unsafe. Others explained actions the healthcare team took to ruin the trust between them, either by not sharing the full truth of the current process or by giving false information. When the providers were not honest with their patients, the birthing person was less likely to feel safe and therefore it tainted their birthing experience with anxious feelings.

#### Subtheme 5C: Feeling in control of the birth

Participants also described feeling in control of the space allowed them to feel safe within the birthing space. Participants who were given the ability to make decisions about positions, movements, and even presented with a way to watch the birth felt in control and supported by staff. Conversely, participants who were restricted in their movement felt trapped.

## Discussion

The findings of this QIMS-DTT highlight what is necessary to have a safe and sacred healing environment for mothers. Filtered through the adapted Theory of Supportive Care Settings, the findings of this deductive theory-testing study found multiple overlaps with the theoretical approach and as such, propose the importance of utilizing a Theory of Supportive Birthing Environments when evaluating birthing care environments. The five main components of Edvardsson’s theory can be found across all included articles and in the findings of this QIMS-DTT, making the findings unique in the application of the theory as a framework to approach environmental birth design.

For instance, a novel finding was the participant-described need for a welcoming birthing environment, including their initial admission to the hospital, being believed, and validated about their labor process, and the birthing environment itself being welcoming to them and their support persons. The initial moments upon arrival at the birthing facility or the presence of the healthcare team can significantly impact the birthing person's emotional well-being, comfort, and sense of security. Indeed, research does indicate that a warm welcome can help alleviate these feelings by making the birthing person feel valued, respected, and cared for from the moment they arrive. A positive and supportive atmosphere can contribute to a more relaxed state of mind [47]. Although the findings illuminate that a warm welcome into the birthing environment is critically important as it sets the tone for the entire childbirth experience, there is scant literature on this phenomenon as an attribute of the birth environment experience. A warm welcome also fosters trust and rapport between the birthing person and the healthcare team [46] which is essential for effective communication and cooperation throughout labor and childbirth. When trust is established early on, it can lead to a more collaborative and positive birthing experience. Beyond alleviating stress, feeling welcomed and respected empowers the birthing person to actively engage in their care and decision-making [47]. When they are treated with kindness and dignity, they are more likely to voice their preferences, concerns, and questions, leading to informed decision-making [47, 77]. As many participants shared, the birthing environment itself was responsible for the welcoming feeling and contributed to a positive and comfortable birthing environment. In this study, this included friendly greetings, a clean and inviting room, soft lighting, and soothing sounds. Such an environment can promote relaxation and facilitate a smoother labor and birth [77].

The findings also illuminate the importance of social connection within the birthing space, through feeling respected and heard, clear communication, and acknowledgment and validation. Social relationships, including those with partners, family members, friends, and healthcare providers, offer emotional support during a time that can be physically and emotionally challenging. Previous literature has supported these findings, indicating that when there are people who care about the birthing person's well-being and provide comfort and encouragement, it can reduce stress and anxiety for the birthing person [40]. Trust is a critical component of any healthcare relationship, especially during childbirth [52]. Unique within these findings, however, is the importance of social connection between the women and providers on the recounting of birth stories and satisfaction with the birth environment. Furthermore, although support by providers is well documented, the findings here offer a unique approach as establishing these relationships as a facet of the birth environment. Establishing trust with healthcare providers and support staff is essential for effective communication, which, in turn, leads to better decision-making and a more positive birthing experience.

Safety in the environment was a salient finding of this study, and with good reason. Participants expressed that having interpersonal safety, seeing a good team effort among healthcare providers, and confidence in that healthcare team all contributed to their perceptions of safety in the birthing environment. Creating feelings of safety in the birthing environment is of paramount importance for several reasons. A safe and supportive birthing environment not only ensures the physical well-being of the birthing person and baby but also has a profound impact on the overall childbirth experience. Feelings of safety help reduce stress and anxiety during labor and childbirth [78]. Perceived safety benefits medical providers as well- when the birthing environment is perceived as safe, it can facilitate the release of endorphins, the body's natural pain relief hormones, and contribute to a smoother labor and birth process without unnecessary medical interventions [79].

Another important, but already substantiated, finding within safety in the environment was the element of control and agency within the birthing environment that was necessary to have positive birth experiences. Participants engaged in self-advocacy and described the importance of feeling in control over the birthing process to their well-being. Agency and control in the birthing environment are documented crucial aspects of the childbirth experience, as they can significantly impact the physical and emotional well-being of the birthing person and their overall satisfaction with the process [45]. When birthing people have a sense of agency and control over their birth experience, they report higher levels of satisfaction with the process, regardless of whether their birth unfolds as planned or not [45]. Agency and control also empower the birthing person to make informed decisions about their birth plan and medical interventions and endorse their maternal role. Informed decision-making allows individuals to choose the options that align with their values, preferences, and health needs. Notably, the findings in this study indicate that when birthing persons do not feel in control of their birth, they had poor retrospective memories about their birth and sometimes felt shame or anger about it. Indeed, a lack of agency and control during childbirth can sometimes lead to feelings of trauma or dissatisfaction [80]. Although this phenomenon is well documented, the findings from this review contextualize the need for agency and control within the theoretical approach and creates a more comprehensive look at birth environment attributes.

### Implications for providers and research

The findings of this study illuminate numerous implications for providers and researchers. For providers, the knowledge that a warm welcome extends beyond them to the entire birthing team, including nurses, midwives, doulas, and support persons. A cohesive and supportive team that welcomes the birthing person with open arms can enhance the overall birthing experience. Furthermore, welcoming includes initial contact and the way a birthing person is received and treated upon arrival can significantly influence their overall perception of their birth experience. A warm welcome contributes to positive birth memories and can have long-lasting emotional and psychological benefits [47].

Empowering birthing people to have control over their experience can help reduce the risk of trauma. Establishing trust and effective communication between the birthing person, their support team, and healthcare providers is essential for maintaining agency and control. When there is open dialogue and mutual respect, the birthing person is more likely to feel comfortable expressing their preferences and concerns. In some cases, having control over the birthing environment can lead to better physical outcomes. For example, a birthing person who can move freely, choose their birthing position, and have access to comfort measures may experience shorter labor and fewer complications [77]. In addition, providers should recognize that every birthing experience is unique and respecting cultural and individual differences is essential for promoting agency and control. What one person values or finds empowering in their birthing experience may differ from another, and healthcare providers should strive to accommodate these variations. More research may be needed to understand the prevalence of agency and control better quantitatively in the birthing environment and its relationship to maternal mental health outcomes using measurements surveying the birth environment that combine the attributes of the framework presented in the findings.

Building social relationships in the birthing environment can create a supportive and celebratory atmosphere. The birthing person, their partner, and their support network can share in the joy and excitement of welcoming a new life into the world, enhancing the overall experience.

Social relationships formed during childbirth can extend into the postpartum period, providing ongoing emotional support, advice, and assistance as the birthing person navigates the challenges of early parenthood. Social relationships in the birthing environment can also be a source of valuable information and education. Healthcare providers and support persons can share knowledge about the birthing process, available options, and potential interventions, empowering the birthing person to make informed decisions.

Another implication for providers is building a culture of safety within the environment. When the birthing environment feels unsafe or traumatic, it can have long-lasting negative effects on the birthing person's mental and emotional well-being. Feelings of trauma during childbirth can lead to post-traumatic stress disorder [PTSD] and have a significant impact on future pregnancies [80, 81]. Safety also includes trust. Trust is a cornerstone of the birthing experience and when the birthing person trusts their healthcare providers and the birthing environment, they are more likely to follow recommendations, cooperate with care plans, and have a positive overall experience. More research is needed to better understand how women experience trust in the birthing environment specifically, including better understanding of the frequencies of agency, consent, and control over their environments. In addition, research surveying the use of interdisciplinary communication and communication mechanisms with women regarding birth plans might illuminate fragmented communication in the birth environment.

## Limitations

Within this study there were some primary limitations related to sampling of studies. When identifying studies through databases and services such as GoogleScholar, embargoes and artificial intelligence interference [e.g., search algorithms] create challenges in replicating and updating searches. For this study, the search was initially conducted then redone to ensure all studies were identified since sufficient time had passed since the initial search. Although exact keywords and procedures were followed from search one to search two, algorithms and embargoes may have led to some key studies not emerging in the search. A second limitation is that given the breadth of birthing environments and cultural orientations to birthing, despite the number of studies analyzed, it is likely that some experiences are not represented in this study.

While the experiences of the participants appeared to range, the scope of the search did not include birthing person experiences outside of the US. Consequently, this leaves the results of this study to only be applicable to what is needed in the small context of the US. Problems that are faced by participants in this study may not be seen as harmful to others. Likewise, since QIMS-DTT is a social work focused method, it can limit how the researchers approached the material from the participants. This can be related to the complex nature of constraints that are often faced in the health-care field. Furthermore, there is a limitation related to the relevancy of applying the TSCS to the birthing space. A key difference between the concept of service in birthing space is that mothers only spend an average of 24 to 48 h in the birthing space, whereas those in nursing care, the environmental in which TSCS originated, could spend an extended period of time in the environment.

## Conclusion

In conclusion, a new framework using the Theory of Supportive Care Settings can be applied to evaluate a sacred and healing birthing experience. This new framework includes a balance of already documented phenomenon such as agency and control during birth, as well as integrates new findings, such as the necessity of a warm welcome into the birthing environment to promote trust, comfort, and empowerment. Indeed, the importance of a welcoming environment cannot be overstated. It sets the initial tone for the birthing experience, influencing the individual's stress levels and emotional state, which, in turn, can affect the physiological aspects of childbirth. This study supports the hypothesis from applying TSCS to the birth environment that when individuals feel welcomed, they are more likely to experience a sense of calm and readiness for birth, which can lead to more positive outcomes.

Our study contributes to the growing body of literature that underscores the significance of the birth environment in shaping birth experiences. It calls for a reevaluation of current practices and environments in which childbirth takes place, advocating for a more holistic approach that encompasses emotional, psychological, and physical well-being. The implications of our findings extend beyond the individual, suggesting that by improving birth experiences, we can foster better early bonding experiences, potentially leading to long-term benefits for both the mother and child.

## Authors’contributions

Authors DM and SL contributed to the initial design and concept. DM, SL, RT, and TG all performed data collection, data analysis, interpretation of results, and drafting of the article. All authors made substantial contributions to the initial and revised manuscript. All authors have read and approved the final version and are accountable for all aspects of the work.

## Data Availability

The data used in this study are from publicly available existing literature, therefore the data is available within this article from the data tables.
